# Changes in the urinary metabolome accompanied alterations in body mass and composition in women with overweight – impact of high versus low protein breakfast

**DOI:** 10.1007/s11306-024-02156-5

**Published:** 2024-07-27

**Authors:** Banny Silva Barbosa Correia, Line Barner Dalgaard, Line Thams, Mette Hansen, Hanne Christine Bertram

**Affiliations:** 1 Department of Food Science, Aarhus University, Agro Food Park 48, Aarhus N, 8200 Danmark; 2 Department of Public Health, Aarhus University, Aarhus, Denmark

**Keywords:** Weight loss, Protein-rich diet, Proton NMR spectroscopy, Metabolic phenotype, N, N-dimethylglycine, Trigonelline, Trimethylamine

## Abstract

**Introduction:**

Understanding why subjects with overweight and with obesity vary in their response to dietary interventions is of major interest for developing personalized strategies for body mass regulation.

**Objectives:**

The aim of this study was to investigate the relationship between changes in the urine metabolome and body mass during a breakfast meal intervention. Furthermore, we aimed to elucidate if the baseline urine metabolome could predict the response to the two types of breakfast meals (high versus low protein) during the intervention.

**Methods:**

A total of 75 young, women with overweight were randomly allocated to one of two intervention groups: (1) High-protein (HP) or (2) low-protein (LP) breakfast as part of their habitual diet during a 12-week intervention. Beside the breakfast meal, participants were instructed to eat their habitual diet and maintain their habitual physical activity level. Nuclear magnetic resonance-based metabolomics was conducted on urine samples collected at baseline (wk 0), mid-intervention (wk 6), and at endpoint (wk 12). At baseline and endpoint, body mass was measured and DXA was used to measure lean body mass and fat mass.

**Results:**

The baseline urine metabolite profile showed a slightly higher correlation (R2 = 0.56) to body mass in comparison with lean body mass (R2 = 0.51) and fat mass (R2 = 0.53). Baseline 24-h urinary excretion of trigonelline (*p* = 0.04), N, N-dimethylglycine (*p* = 0.02), and trimethylamine (*p* = 0.03) were significantly higher in individuals who responded with a reduction in body mass to the HP breakfast.

**Conclusions:**

Differences in the urine metabolome were seen for women that obtained a body weight loss in the response to the HP breakfast intervention and women who did not obtain a body weight loss, indicating that the urine metabolome contains information about the metabolic phenotype that influences the responsiveness to dietary interventions.

**Supplementary Information:**

The online version contains supplementary material available at 10.1007/s11306-024-02156-5.

## Introduction

Globally, obesity is an increasing challenge for human health. Thus, WHO recently released a report on the state of the obesity pandemic in Europe, which revealed that 60% of citizens in Europe are either with overweight or with obesity (WHO, 2022), and the World Obesity Atlas predicts that 1 out of 5 women, and 1 out of 7 men will be obese in 2030 worldwide (WOA, 2022). A sedentary lifestyle with minor physical activity as well as easy access to food all day are some of the main driving factors. Many dietary interventions have been attempted with varying success and large inter-individual responses. However, several cross-sectional studies report that eating breakfast is associated with a lower body mass index (BMI) and lower incidences of obesity compared to not eating breakfast (Horikawa et al., 2011; Ma et al., 2020). The underlying mechanisms establishing an association between breakfast intake with regulation of BMI are probably related to satiation effects modifying energy intake during the day. Intriguingly, protein-rich meals have been shown to give greater satiety (Veldhorst et al., 2008), and in a recent study it was shown that protein-rich breakfast was associated with high satiety compared to an isocaloric breakfast with lower protein content or no breakfast in young women with overweight (Dalgaard et al., 2023). Therefore, we recently conducted a 12-week intervention study to investigate the effects of consuming a dairy-based, high-protein breakfast (HP) or an isocaloric low-protein (LP) breakfast for 12 weeks on body composition in 18-30-year-old women with overweight or with obesity. We hypothesized that a high compared with a low protein breakfast would induce enhanced satiety and reduce daily energy intake and thereby lower fat mass and waist circumference (WC). However, we observed a tremendous inter-individual variation in the response to diet interventions (Dalgaard et al., 2023). Both for subjects consuming a dairy-based, HP breakfast and for subjects consuming an LP breakfast, the intervention resulted in both body mass gain and body mass loss among the subjects (Dalgaard et al., 2023). Thus, some participants gained several Kg of body mass while some participants lost 7 Kg of body mass. The reason for this inter-individual variation remains unknown.

The metabolome is considered a signature of our metabolic state. Thus, it has been proposed that the metabolome offers a dynamic, comprehensive, and precise picture of the human phenotype (Gonzalez-Covarrubias et al., 2022). Accordingly, serum metabolomics profiling has been employed to study associations between the metabolome and obesity. Among the largest studies reported is a cross-sectional study of 947 participants, which found that 37 metabolites were significantly correlated with BMI (Moore et al., 2014). However, research on the metabolites that are related to changes in body mass or fat mass, is limited (Vijay & Valdes, 2019). We hypothesized that the urine metabolome might provide insight into inter-individual variations in the response to a dietary intervention. Thus, in the present study, the metabolome of 24-h urine samples collected at baseline, after 6 weeks and 12 weeks of a dietary intervention with HP or LP breakfast was analyzed to determine (i) if the metabolome at baseline could predict the response to a breakfast intervention, and (ii) if relations existed between changes in the metabolome and the change in body mass during the intervention. The urine metabolome was analyzed as it is easily accessible and represents the biofluid with highest number of detectable metabolites (Bouatra et al., 2013).

## Materials and methods

### Sample collection and study design

The study design has previously been described by Dalgaard et al. (Dalgaard et al., 2023). Briefly, 75 young women with overweight (BMI ≥ 25 kg/m^2^) were enrolled in the study. During a 12-week intervention, the women were allocated to one of two groups where they were instructed to daily consume either (1) HP, or (2) LP breakfast meal. Twenty-four h urine samples were collected at week 0 (wk-0) (baseline), at week 6 (mid-intervention) and at week 12 (wk-12) (endpoint). After sampling, samples were frozen and stored at -80 ºC until further analysis. Body mass was measured by using a digital scale, and lean body mass and fat mass were measured by DXA scanning at baseline and endpoint.

The study was conducted in accordance with Good Clinical Practice and the study protocol complied with the relevant sections of the Declaration of Helsinki. The study was approved in accordance with the standards of the local ethical committee of the Central Denmark Region (MJ-1-10-72-220-19) and was registered at www.clinicaltrials.gov (ID: NCT04518605). The study was conducted from August 2020 to January 2022 at the Section for Sport Science, Department for Public Health, Aarhus University, Denmark.

### Urine metabolites analysis

Urine samples were thawed on ice and gently inverted a couple of times, centrifuged for 5 min at 2000 RCF at 4 °C. For each sample 900 µL of urine 100 uL of buffer for IVDr (deuterium oxide phosphate buffer 0.10 M, pD = 7.4 containing 0.03% of 3-(trimethylsilyl)propionic-2,2,3,3-d4 acid, sodium salt (TSP d4) and 0.04% of sodium azide) was added to an Eppendorf tube, then the tubes were mixed for 1 min at a table mixer at 1400 rpm, and then centrifuged for 1 min at 2000 RCF at room temperature. A volume of 600 µL of the solution was transferred to a 5 mm NMR tube.

NMR spectra were conducted at 300 K on a 600 MHz Bruker Avance III spectrometer equipped with a 5 mm BBI probe and fitted with the Bruker SampleJet robot cooling system. Proton (^1^H) NMR spectra were acquired using the Bruker In Vitro Diagnostics research (IVDr) methods. The NMR instrument was calibrated before the analysis and the automated methods were executed on each sample. For each sample, two experiments were conducted in automation mode, amounting to a total of 17 min acquisition time per sample: a standard one-dimensional (1D) experiment with solvent presaturation, and a two-dimensional (2D) − J-resolved experiment.

The free induction decays (FIDs) were multiplied by a 0.3 Hz exponential function prior to Fourier transformation. Phase and baseline corrections were carried out, the reference standard TSP-d4 signal was adjusted to δ 0.00, and the spectra were assigned using both Chenomx database values and information obtained from 2D NMR spectra. For quantification, Chenomx software was used to integrate and quantify the metabolite peaks relative to the TSP-d4 standard.

### Statistical analysis

Individuals who lost body mass during the 12-wk intervention within the 95% confidence interval were classified as responders while individuals who gained body mass within the 95% confidence interval during the 12-wk intervention were classified as non-responders prior to statistical analysis. A scheme that illustrates the number of individuals, their samples collected, and data acquired is summarized in Figure S1. In summary, data from a total of 57 individuals of the 75 women enrolled in the study completed the intervention and were included in the analysis. Thus, data from 168 urine samples collected at wk-0, wk-6, and wk-12 periods were included in the statistical analyses to access information on urine metabolite profiles.

Univariate analyses were conducted using GraphPad Prism 9.4.1 software (GraphPad Software, Inc.). The univariate method employed two-way ANOVA followed by Tukey’s post hoc tests to compare the mean values between intervention groups. A *p*-value of 0.05 was considered the significant threshold. Data were analyzed using mixed effect models to analyze the main effect of time (wk-0, Wk-6 and wk-12), diet (HP- or LP breakfast meal), and a response to the intervention (weight loss as a positive response) including the interactions effects of time-by-diet, time-by-response, diet-by-response, and time-by-diet-by-response.

Multivariate data analysis was performed by using SIMCA 17 software (Sartorius, Umeå, Sweden). Unit variance scaling was applied to scale the variables before Partial Least Squares (PLS) regression analysis. Pearson correlation, using GraphPad Prism 9.4.1 software (GraphPad Software, Inc.), computed correlation between the phenotype (body mass and composition changes) and every metabolite. Pathway Analysis of urine metabolites was conducted, which used continues input data (regression) with weight change being the phenotype, the Homo sapiens KEGG as the set library through MetaboAnalyst software.

## Results

### Correlation between urine metabolome at baseline and succeeding changes in body mass and composition

**Fig. 1:**
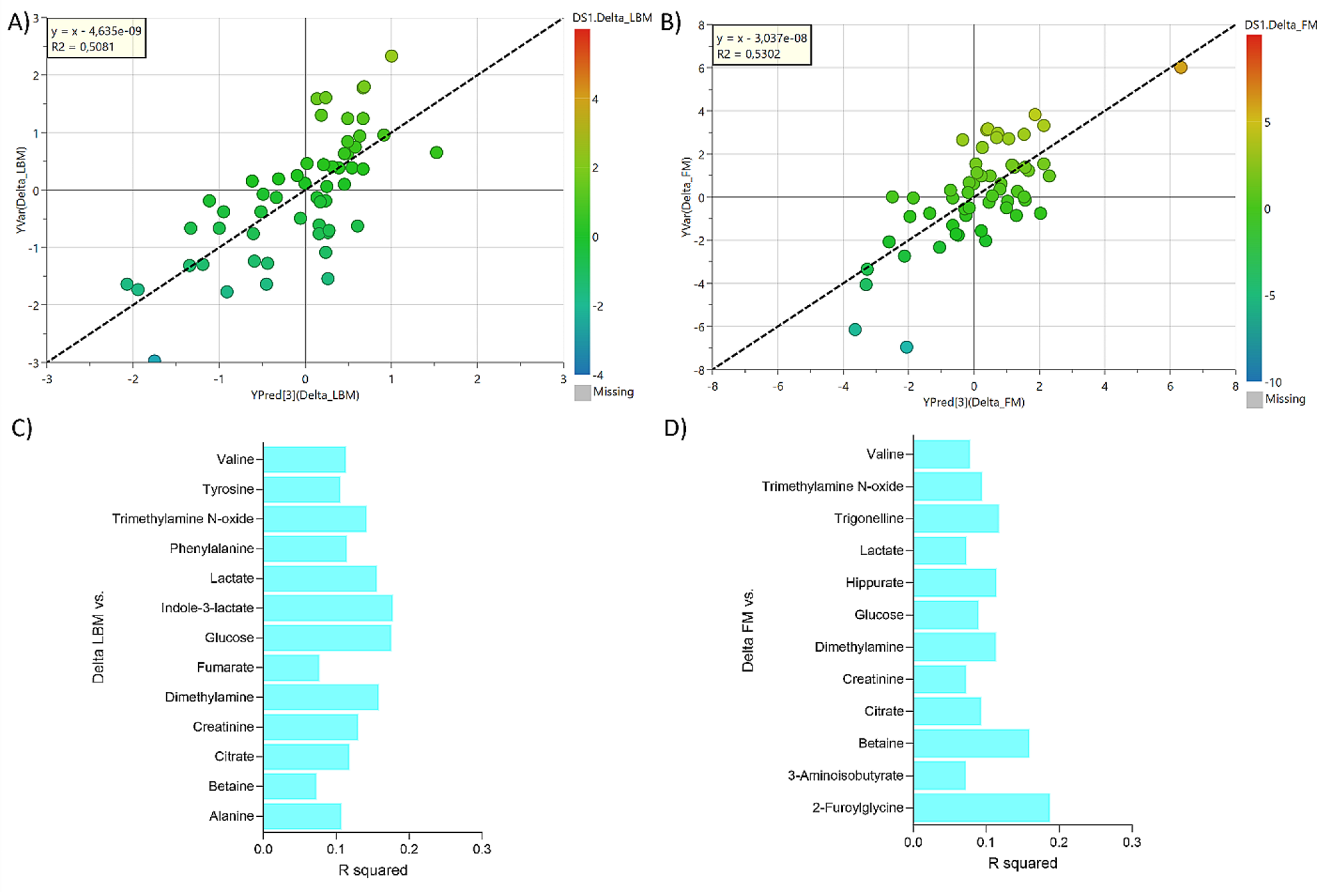
The observed versus predicted plot of PLS models and their Variable Importance for the Projection (VIP) to predict changes in lean body mass (LBM) **(A, C)** and changes in fat mass (FM) **(B, D)** from urine metabolite on baseline samples. An overview of the PLS model validations is provided in Figure S2

**Fig. 2:**
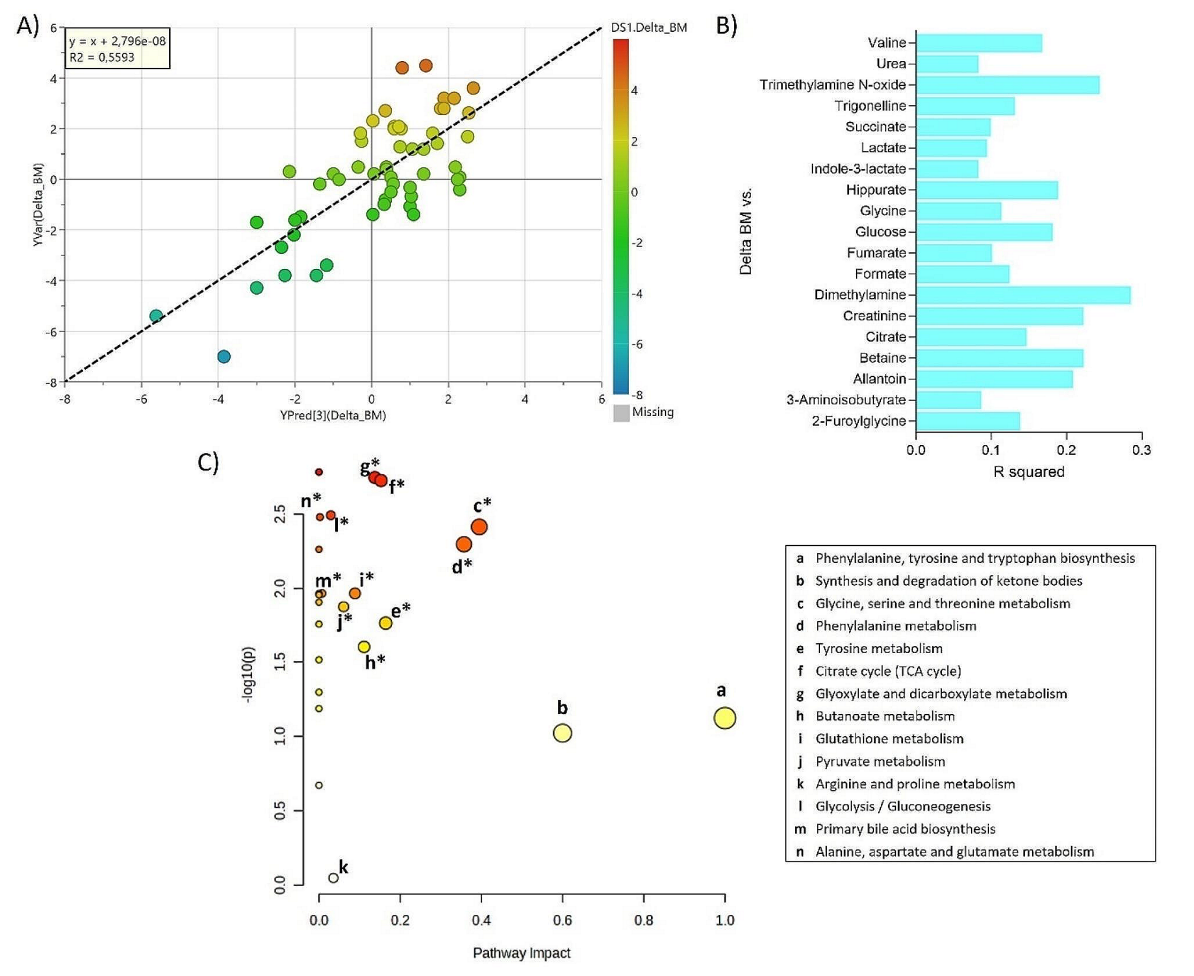
The observed versus predicted plot of PLS model constructed to predict body mass (BM) changes from the urine metabolite profile measured at baseline **(A)**, their Pearson Correlation (VIP) **(B)** with *p*-value ≤ 0.05, and their pathway analysis **(C)** in which the node color reflects *p*-value and the node radius is determined based on their pathway impact values, * indicates *p*-value ≤ 0.05. An overview of the PLS model validation is shown in Figure S3

In total, 31 quantifiable metabolites were detected and identified in the urine samples. To investigate a potential link between the urine metabolite profile and changes in body mass and composition during the breakfast intervention, PLS models were constructed between urine metabolites (measured at baseline) and body mass and composition changes following the 12-wk longitudinal intervention (Fig. 1and Fig. 2).

The baseline urine metabolite profile showed correlation to lean body mass and fat mass (Fig. 1A-B) and evaluation of the correlation coefficients revealed that the metabolites valine, trimethylamine N-oxide (TMAO), lactate, glucose, dimethylamine, creatinine, citrate, and betaine were important for the correlation (Fig. 1C-D).

The results revealed a slightly higher correlation between baseline urine metabolite profile and body mass (Fig. 2A), possibly as a result of a higher variation in body mass changes in comparison with changes in lean body mass (Fig. 1A) and fat mass (Fig. 1B). The corresponding Pearson correlation plot revealed urine metabolites that correlated with the observed body mass changes, which showed that 19 metabolites (2-furoylglycine, 3-aminoisobutyrate, allantoin, betaine, citrate, creatinine, dimethylamine, formate, fumarate, glucose, glycine, hippurate, indole-3-lactate, lactate, succinate, trigonelline, TMAO, urea, and valine) were important metabolites contributing to predict body mass changes (Fig. 2B).

### Metabolic differences between responders and non-responders to the two breakfast diet interventions

Table 1 shows characteristics of the study participants identified as responders and non-responders. No significant differences were found in baseline characteristics of the body mass responders and non-responders except for percentage body fat (%Fat) and blood triglycerides in participants consuming the high protein breakfast.

**Table 1:** Baseline characteristics of the BM responders (≥ 1% weight loss) and non-responders (< 1% weight loss) following high protein (HP) and low protein (LP) breakfast diet for 12 weeks. *P*-value ≤ 0.05 indicates significant differences

	HP			LP		
	Non-responders (*n* = 8)	Responders (*n* = 17)	*P* value	Non-responders (*n* = 11)	Responders (*n* = 20)	*P* value
Age y	26.0	24.5	0.1811	24.6	25.6	0.3759
**Anthropometry**						
BMI	31.2	29.4	0.3980	32.0	33.0	0.6409
Waist	94.7	89.0	0.1602	96.2	94.7	0.7407
%Fat	45.6	39.5	0.0032	43.7	43.1	0.7833
**Glycemic markers**						
Glucose mM	5.4	5.5	0.5968	5.4	5.3	0.4609
Glucose Tolerance Test _120	7.4	6.3	0.1347	7.0	7.7	0.1080
**Lipid profile markers**						
Triglycerides mM	1.5	0.8	0.0012	1.3	1.2	0.6834
Total cholesterol mM	4.8	4.3	0.2667	4.7	4.6	0.8473
HDL cholesterol mM	1.3	1.4	0.7903	1.5	1.3	0.1878
LDL cholesterol mM	2.5	2.7	0.4470	2.7	2.8	0.8072

**Fig. 3:**
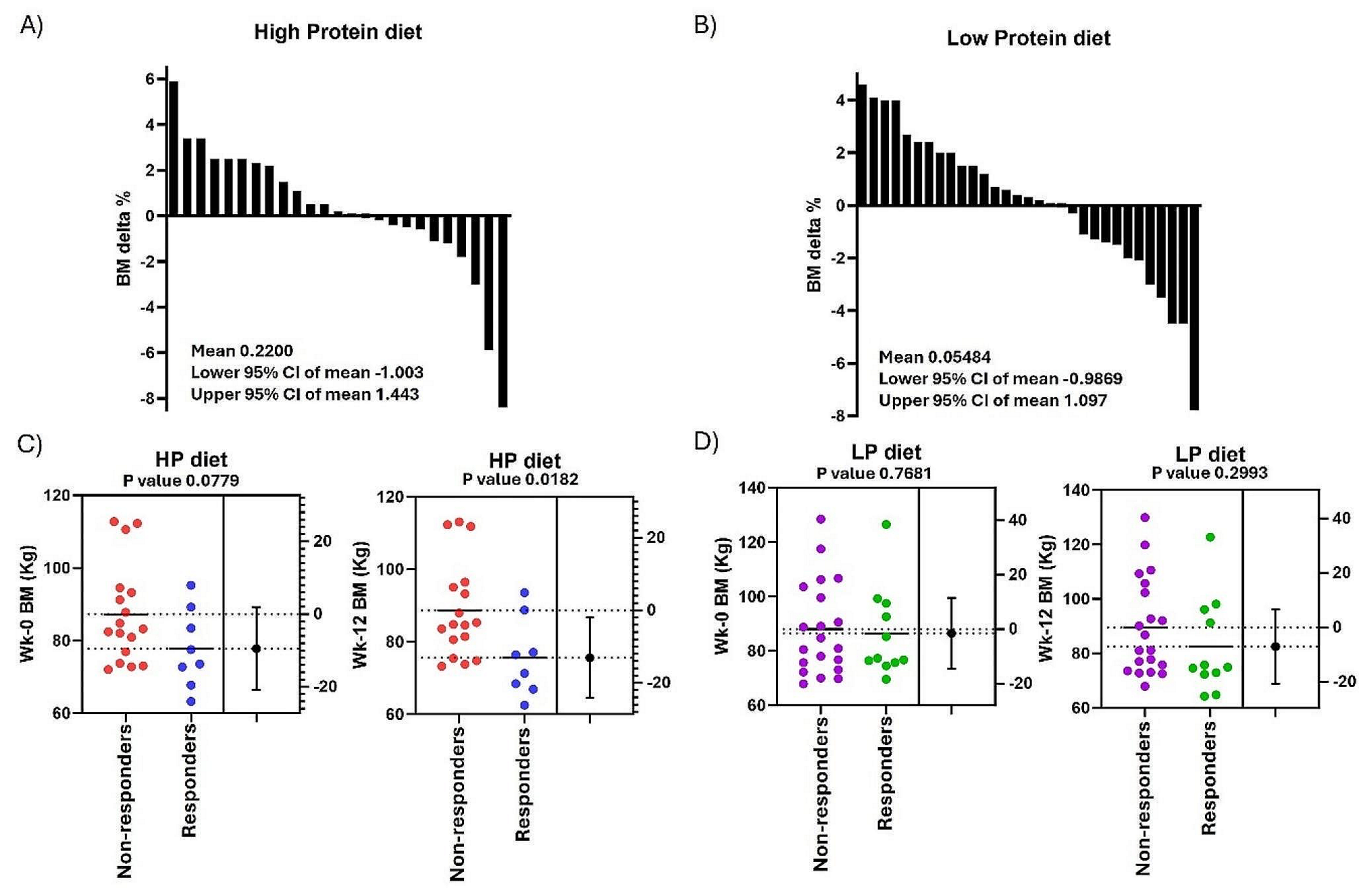
Percentage body mass (BM) change of participants completing 12 weeks intervention following high protein **(A)** and low protein **(B)** breakfasts, respectively. Body mass (BM) in Kilograms by week 0 (baseline), and week 12 (post-intervention), for non-responders and responders to interventions with high protein (HP) **(C)** and low protein (LP) **(D)** breakfasts, respectively. *P*-value ≤ 0.05 indicates significant differences in T-test

According to registered body mass change in high protein and low protein breakfasts (Fig. 3A-B), responders had a weight loss of ≥ 1% and non-responders had a weight loss of < 1%. Body mass change in week 12 (post-intervention) was significantly different between responders and non-responders when intervention with high protein breakfast was evaluated (*p* = 0.018). However, for low protein breakfast (Fig. 3C-D), the body mass change was not significantly different between responders and non-responders (*p* = 0.30).

### Changes in the urine metabolome during high protein versus low protein breakfast intervention

Univariate statistical analysis revealed an effect of time for TMAO, and an effect of diet for 2-furoylglycine, acetate, hippurate, succinate, and trigonelline (Table 2). An effect of response in body mass change to intervention was observed for several metabolites including 3-hydroxybutyrate, acetoacetate, acetone, betaine, citrate, creatinine, dimethylamine, hippurate, indole-3-lactate, phenylalanine, succinate, trimethylamine, TMAO, and urea. In addition, the interaction effect between time and response had *p* values < 0.05 observed for 3-aminoisobutyrate, hippurate, trimethylamine, and trimethylamine N-oxide; while an interaction effect between diet and response was found for acetate, hippurate, and succinate.

**Table 2:** Urine metabolite effects. *P*-value ≤ 0.05 indicates significant differences

	Effects (*P* values)									
	Time	Diet	Change in BM*	Time x Diet	Time x Change in BM	Diet x Change in BM		Time x Diet x Change in BM		
**1-Methylhistidine**	0.12	0.68	0.62	0.90	0.48	0.48		0.50		
**2-Furoylglycine**	0.36	**0.03**	0.29	0.64	0.28	0.22		0.83		
**3-Aminoisobutyrate**	0.13	0.77	0.28	0.53	**0.04**	0.13		0.81		
**3-Hydroxybutyrate**	0.35	0.56	**0.03**	0.38	0.28	0.68		0.59		
**Acetate**	0.25	**0.01**	0.05	0.30	0.73	**0.02**		0.22		
**Acetoacetate**	0.33	0.96	**0.01**	0.20	0.34	0.43		0.33		
**Acetone**	0.41	0.59	**0.04**	0.20	0.40	0.50		0.22		
**Alanine**	0.72	0.45	0.65	0.85	0.16	0.89		0.76		
**Allantoin**	0.58	0.91	0.41	0.65	0.05	0.15		0.76		
**Betaine**	0.98	0.86	**0.02**	0.75	0.20	0.33		0.46		
**Citrate**	0.43	0.75	**0.02**	0.78	0.36	0.22		0.71		
**Creatine**	0.50	0.06	0.83	0.84	0.99	0.05		0.53		
**Creatinine**	0.31	0.14	**0.01**	0.42	0.12	0.57		0.82		
**Dimethylamine**	0.17	0.66	**0.01**	0.21	0.10	0.87		0.88		
**Formate**	0.58	0.22	0.57	0.85	0.42	0.64		0.70		
**Fumarate**	0.80	0.07	0.26	0.56	0.20	0.71		0.66		
**Glucose**	0.49	0.60	0.15	0.38	0.13	0.18		0.81		
**Glycine**	0.87	0.06	0.08	0.94	0.18	0.06		0.64		
**Guanidoacetate**	0.90	0.50	0.76	0.71	0.40	0.34		0.79		
**Hippurate**	0.25	**0.05**	**0.03**	0.86	**0.04**	**0.02**		0.98		
**Indole-3-lactate**	0.58	0.21	**< 0.01**	1.00	0.55	0.23	0.81			
**Lactate**	0.42	0.19	0.68	0.23	0.61	0.96	0.91			
**N**,** N-Dimethylglycine**	0.30	0.97	0.15	0.49	0.89	0.72	0.28			
**Phenylalanine**	0.42	0.31	**0.01**	0.70	0.30	0.26	0.15			
**Succinate**	0.33	**0.02**	**0.02**	0.47	0.58	**0.01**	0.27			
**Trigonelline**	0.42	**0.01**	0.05	0.96	0.47	0.06	0.61			
**Trimethylamine**	0.63	0.47	**0.01**	0.92	**0.05**	0.64	0.56			
**Trimethylamine N-oxide**	**0.01**	0.60	**< 0.01**	0.27	**< 0.01**	0.92	0.65			
**Tyrosine**	0.99	0.21	0.90	0.95	0.25	0.28	0.72			
**Urea**	0.68	0.06	**0.01**	0.21	0.76	0.42	0.70			
**Valine**	0.78	0.51	0.06	0.35	0.30	1.00	0.94			

* refers to if participants lost or gained body mass (BM) during the intervention

**Fig. 4:**
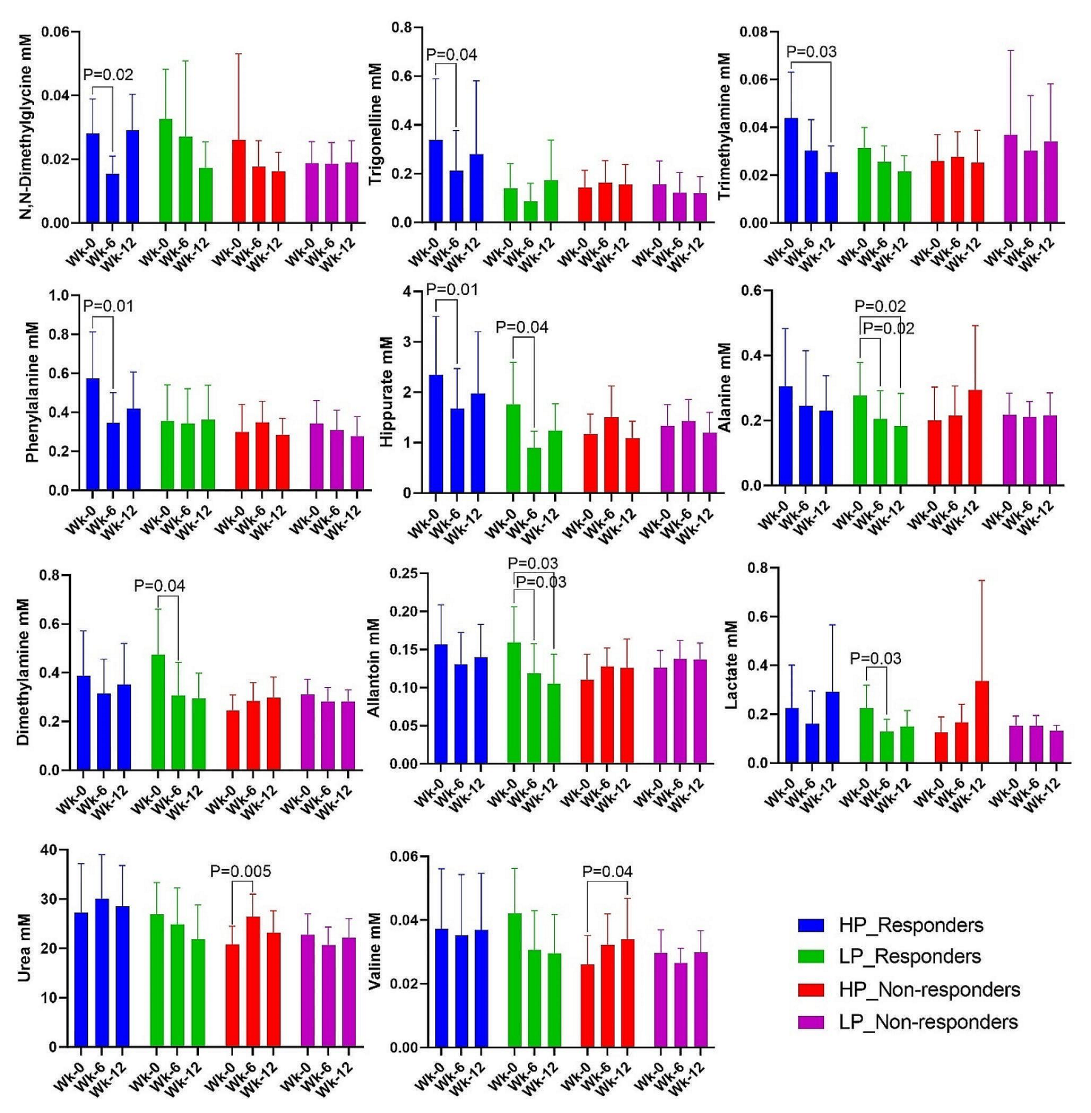
Concentrations of urinary metabolites showing significant differences between individuals obtaining a reduction in body mass (responders) to interventions with high protein (HP) and low protein (LP) breakfasts, respectively. *P*-value ≤ 0.05 indicates significant differences in post-hoc tests within each metabolite comparing the three time points, week 0 (wk-0) (baseline), week 6 (mid-intervention) and week 12 (wk-12) (endpoint). See Table S1 to access complete list of urine metabolite concentrations

N, N-dimethylglycine, trigonelline, trimethylamine, phenylalanine, and hippurate concentrations decreased significantly during intervention with HP diet in women, who lost body mass (HP responders) (Fig. 4); while hippurate together with alanine, dimethylamine, allantoin, and lactate concentrations decreased significantly during LP diet intervention in women, who lost body mass during the intervention (LP responders). Urea and valine levels were significantly increased during HP diet intervention by women, who did not respond with a body mass loss.

## Discussion

The present study aimed to investigate the relationship between the urinary metabolite profile and changes in body mass and composition during an intervention with a HP breakfast meal in young women with overweight in comparison with a LP breakfast meal. Our study revealed that regardless of HP or LP intervention, the urine metabolome at the baseline could predict the body mass, lean body mass, and fat mass changes. In special, body mass changes showed a Pearson correlation with 19 significant metabolites of which 8 (valine, TMAO, lactate, glucose, dimethylamine, creatinine, citrate, betaine) were the same correlating with lean body mass and fat mass changes; and 11 (2-furoyglycine, 3-aminoisobutyrate, allantoin, formate, fumarate, glycine, hippurate, indole-3-lactate, succinate, trigonelline, urea) were exclusively to body mass changes (Figs. 1 and 2). In addition, individuals who responded to the HP breakfast meal as reflected in a reduction in body mass concomitantly had a significant reduction in urinary excretion of N, N-dimethylglycine, trigonelline, trimethylamine, phenylalanine, and hippurate during the intervention (Fig. 4). In contrast, individuals who did not achieve a reduction in body mass as a result of HP breakfast meal intervention did not display these metabolite changes. This finding suggests that differences in endogenous metabolism related to the metabolic phenotype might contribute to variations in the response to an intervention with a HP breakfast in comparison with a LP breakfast.

N, N-dimethylglycine is an amino acid derivative that the human body produces when metabolizing choline into glycine. N, N-dimethylglycine is also a byproduct of homocysteine metabolism where homocysteine and betaine are converted to methionine and N, N-dimethylglycine by betaine-homocysteine methyltransferase. In women, a high concentration of urinary N, N-dimethylglycine has been identified as a predictor of diabetes (Friedrich et al., 2015). In addition, a study examining the association between the urine metabolome and 5-year changes in markers of glucose homeostasis found that N, N-dimethylglycine were among the metabolites showing highest association to glucose homeostasis markers (Fridrich et al., 2018). It has also previously been found to be a useful predictor for progression in knee osteoarthritis (Loeser et al., 2016). The results from these former studies indicate that a reduction in urinary N, N-dimethylglycine is associated with lower diabetes risk and lower inflammatory status. It remains to be established if the beneficial effects can be associated with the involvement of N, N-dimethylglycine in homocysteine metabolism.

Trigonelline is an alkaloid, and high amounts have been found in arabica coffee, fenugreeks and peas. Trigonelline is also a product of the niacin metabolism. Intriguingly, similar to N, N-dimethylglycine, urinary trigonelline was also identified as a predictive marker for diabetes, glucose homeostasis and osteoarthritis in the above-mentioned studies (Friedrich et al., 2015; Loeser et al., 2016; Friedrich et al., 2018). Studies on oral administration of trigonelline have also revealed beneficial effects on insulin resistance and glucose tolerance in both murine models (Yoshinari et al., 2009; Yoshinari & Igarashi, 2010) and humans (van Dijk et al., 2009). While these studies suggest a positive association between trigonelline and glucose metabolism, the present study identified a decrease in urinary trigonelline with a reduction in body mass. However, as the present study identified decreases in trigonelline in participants that lost weight as response to the intervention, the present findings are consistent with a study on obese subjects undergoing gastric bypass, which reported that higher urinary trigonelline levels were associated with an obese phenotype (Calvani et al., 2010). Involvement of the gut microbiota in urinary trigonelline excretion has been proposed (Calvani et al., 2010), but remains to be established.

Trimethylamine (TMA) is a metabolite that can be generated from microbial metabolization of choline, betaine and carnitine. In the liver, TMA is oxidized to trimethylamine-N-oxide (TMAO) by hepatic flavin-containing monooxygenase 3 (FMO3). Studies have linked circulating levels of TMAO with diabetes (Dambrova et al., 2016; Li et al., 2022), and the 5-year longitudinal study by Friedrich et al. (Friedrich et al., 2018) also found a positive association between urinary TMA levels and HbA1c, a diagnostic marker of diabetes. Thus, the observed decrease in urinary TMA excretion for responders to the HP breakfast in the present study might also reflect that the responders experienced an improved glucose homeostasis.

In addition to the three metabolites discussed above, pathway analysis also revealed that endogenous metabolism differed among women that responded to breakfast interventions with a body mass loss and non-responders (Fig. 2). Thus, citrate cycle (TCA) metabolism was found to differ. Intriguingly, a study based on a knowledge discovery from databases proposed that an inhibition of the TCA cycle is a crucial event in the chain of metabolic processes leading to obesity. The inhibition disturbs energy metabolism and results in ATP deficiency with simultaneous fat accumulation (Wlodek & Gonzales, 2003). Glyoxylate/dicarboxylate metabolism was also found to differ between women that responded to breakfast intervention with a body mass loss and non-responders, addressing the important role that glycine seems to play in body mass loss. Proffit et al. have shown that glyoxylate/dicarboxylate metabolism is upregulated in metabolic disorders (obesity, Type 2 diabetes, and atherosclerosis cardiovascular disease) (Proffitt et al., 2022), while Song proposed a relationship between glyoxylate pathway and fat-induced hepatic insulin resistance (Song, 2000).

## Conclusion

In conclusion, the present study revealed that women with overweight, who reduced body mass in response to an intervention with a HP breakfast had a different change in the urine metabolome than women who did not lose body mass. While regression analyses revealed several metabolites to be involved in the correlation between the urine metabolome and body mass changes, univariate statistical analysis suggested that only the women in the HP group who lost body mass experienced a reduction in the metabolites N, N-dimethylglycine, trigonelline, TMA, phenylalanine, and hippurate. Pathways analysis implied that the differences in metabolic phenotype were related to endogenous metabolism involving the TCA cycle and glyoxylate/dicarboxylate metabolism. Thus, using NMR-based characterization of the urine metabolome, new insight into specific differences in the metabolic phenotype linked to a reduction in body mass in response to a HP breakfast intervention was obtained.

### Electronic supplementary material

Below is the link to the electronic supplementary material.

Supplementary Material 1

## Data Availability

Data reported in this paper will be made available upon request.
