# Supplementary material for: Changes in the urinary metabolome accompanied alterations in body mass and composition in women with overweight – impact of high versus low protein breakfast
Source: Metabolomics. 2024 Jul 27;20(4):81. doi: 10.1007/s11306-024-02156-5 (PMC11283391; doi:10.1007/s11306-024-02156-5)
Supplement: Supplementary file 1 — Supplementary Material 1 [file 11306_2024_2156_MOESM1_ESM.docx]

**Supporting Information**


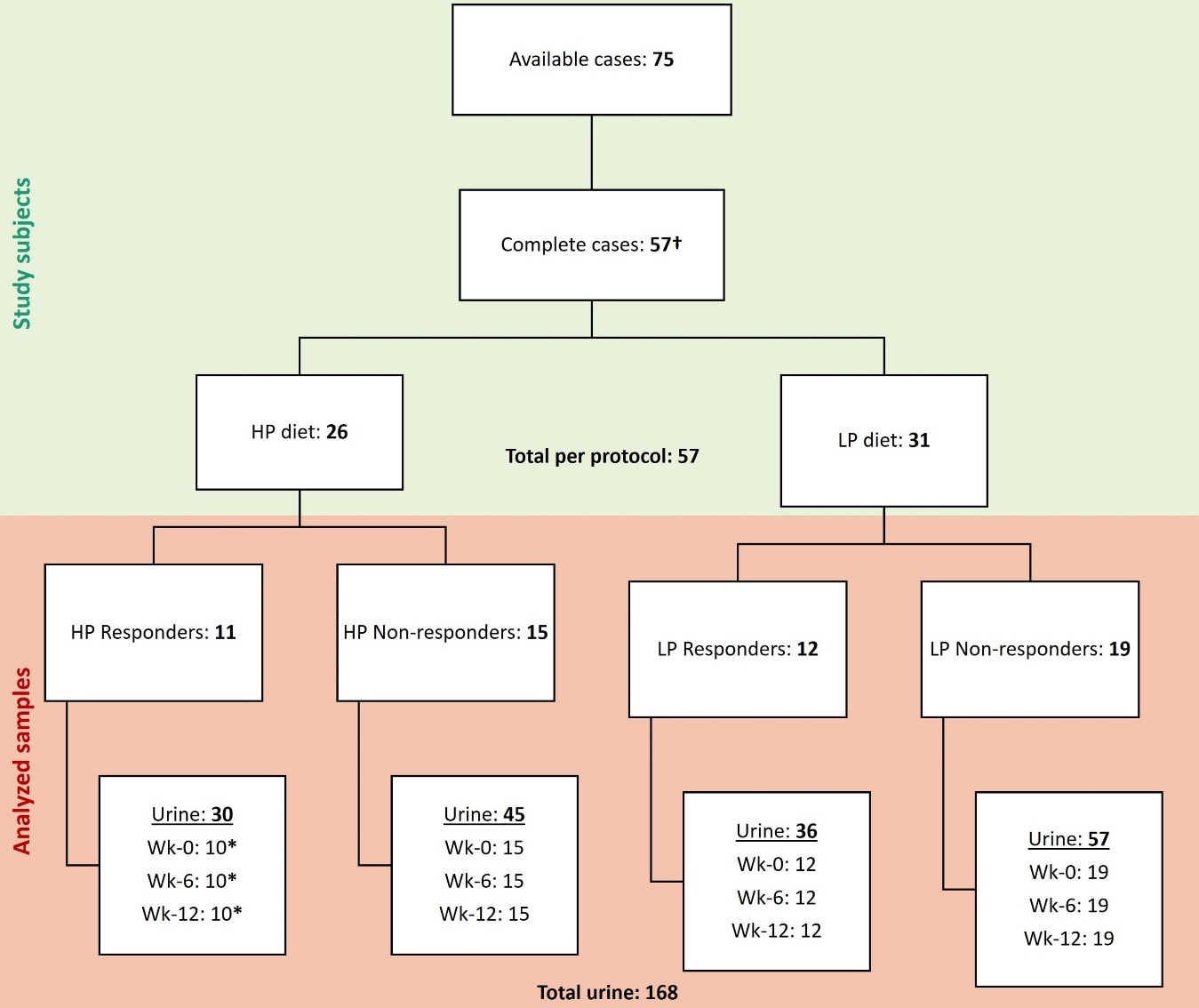


**Fig S1.** Scheme of samples used in analyses of urine metabolites from samples collected at week 0 (wk-0) (baseline), at week 6 (mid-intervention) and at week 12 (wk-12) (endpoint). †Eighteen individuals had all samples collected but dropped out of the clinical measurements. * One sample excluded due to insufficient sample material.


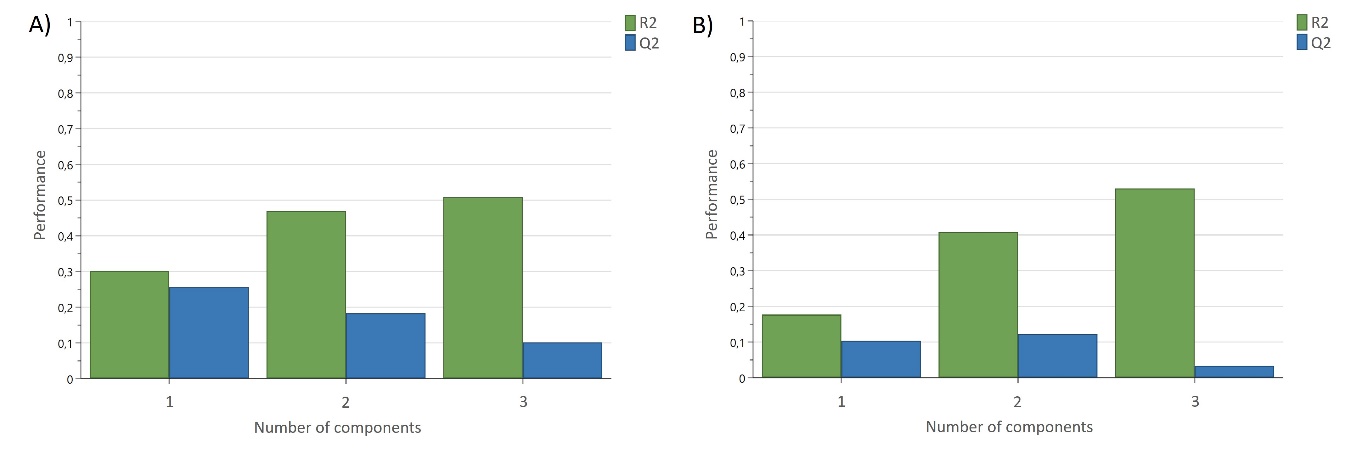


**Figure S2.** Overview of the model validation of the PLS model to predict changes in lean body mass (LBM) (A) and fat mass (FM) (B) from urine metabolite on baseline samples. Cumulative values of R2 (50.8% for LBM, 53.0% for FM) and Q2 (18.3% for LBM, 12.2% for FM) are shown.


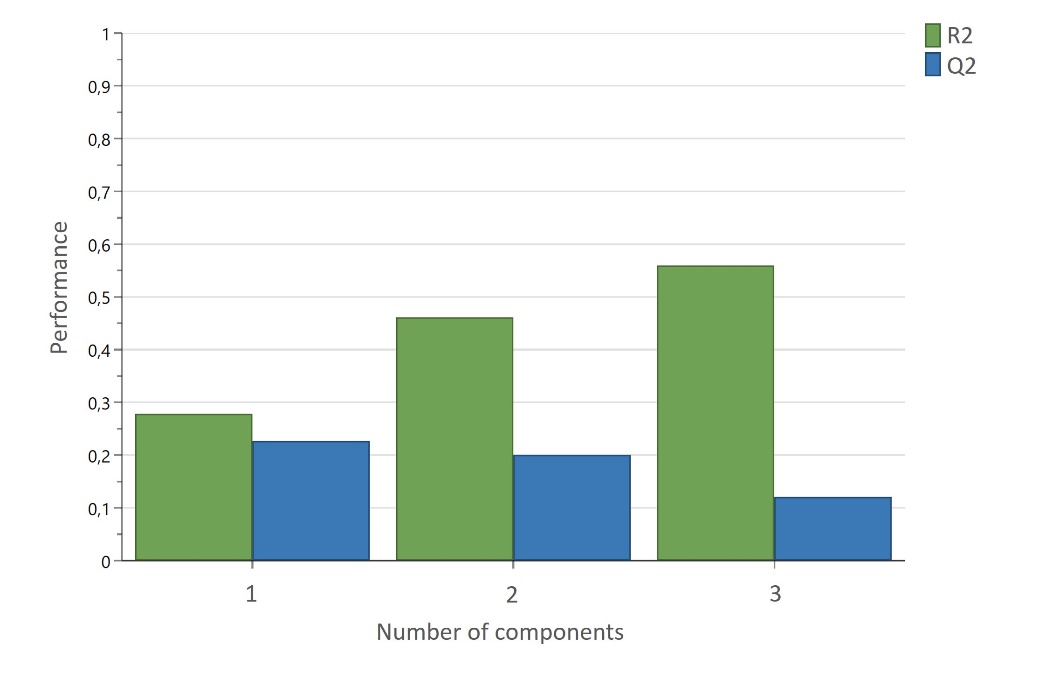


**Figure S3.** Overview of the model validation of the PLS model to predict body mass (BM) change from urine metabolite on baseline samples. Cumulative values of R2 (55.9 %) and Q2 (20.0 %) are shown.


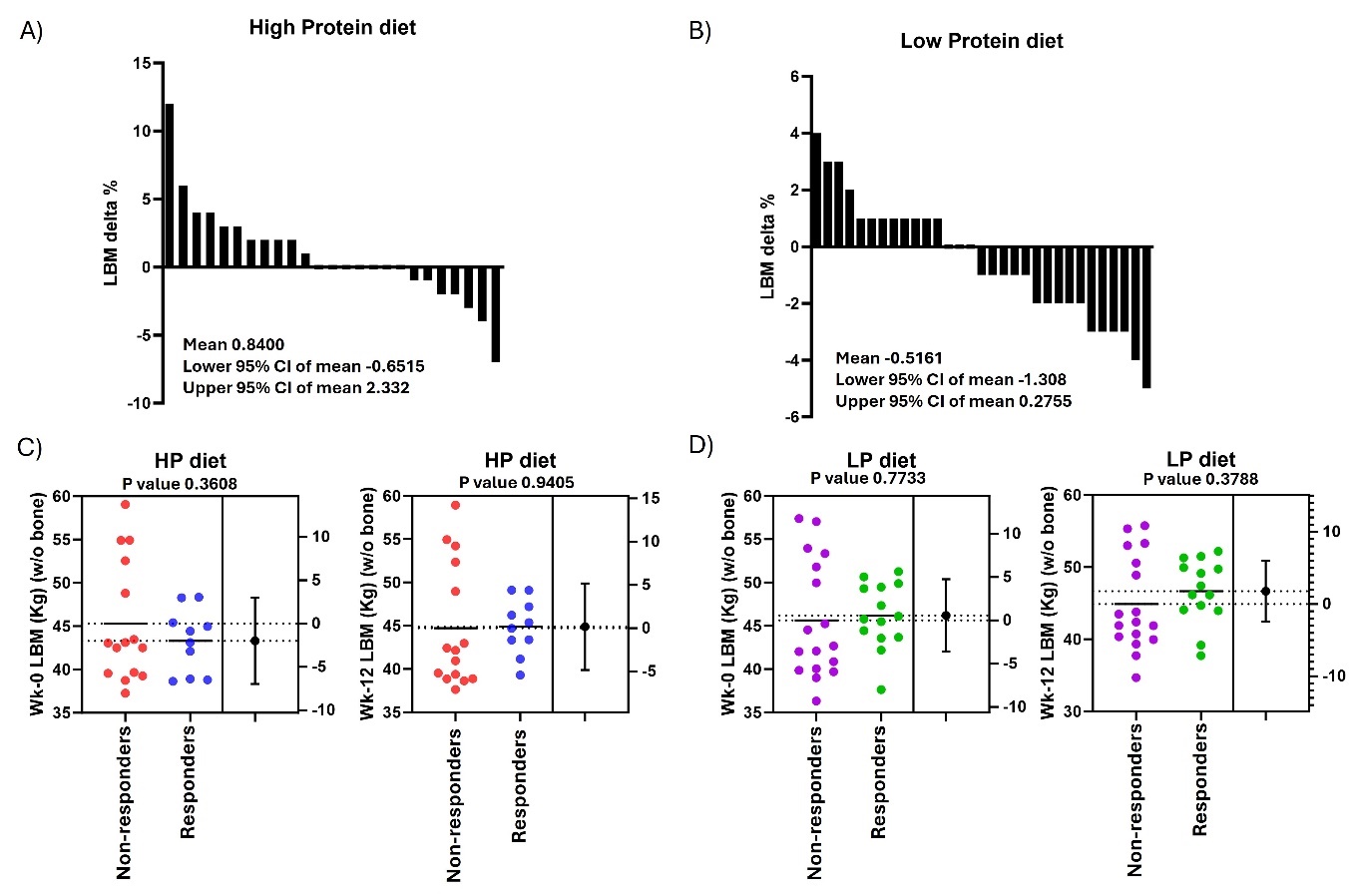


**Figure S4.** Percentage lean body mass (LBM) change of participants completing 12 weeks intervention following high protein (A) and low protein (B) breakfasts, respectively. Lean body mass (BM) in Kilograms by week 0 (baseline), and week 12 (post-intervention), for non-responders and responders to interventions with high protein (HP) (C) and low protein (LP) (D) breakfasts, respectively. P-value ≤ 0.05 indicates significant differences in T-test.


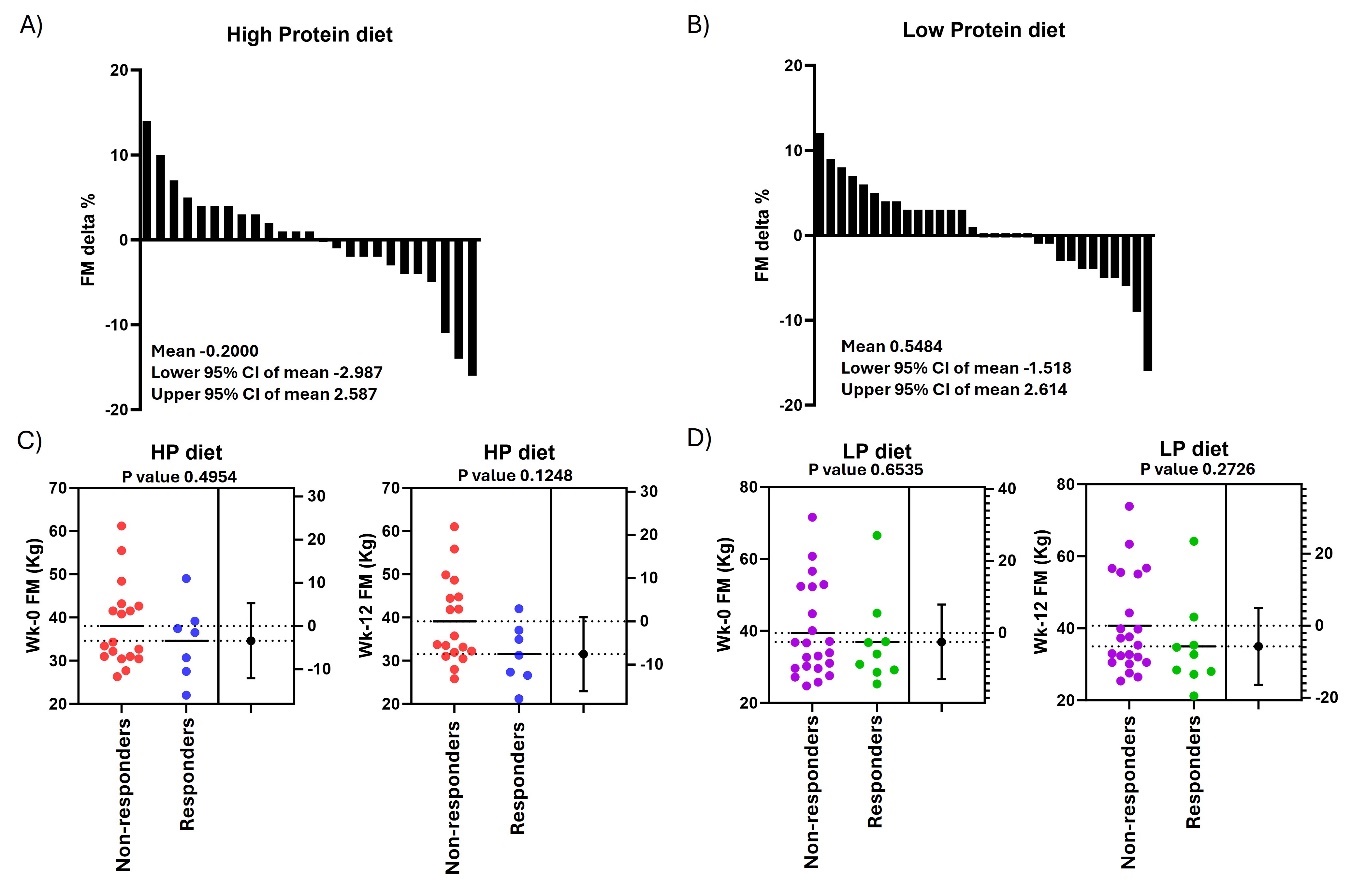


**Figure S5.** Percentage fat mass (FM) change of participants completing 12 weeks intervention following high protein (A) and low protein (B) breakfasts, respectively. Fat mass (FM) in Kilograms by week 0 (baseline), and week 12 (post-intervention), for non-responders and responders to interventions with high protein (HP) (C) and low protein (LP) (D) breakfasts, respectively. P-value ≤ 0.05 indicates significant differences in T-test.


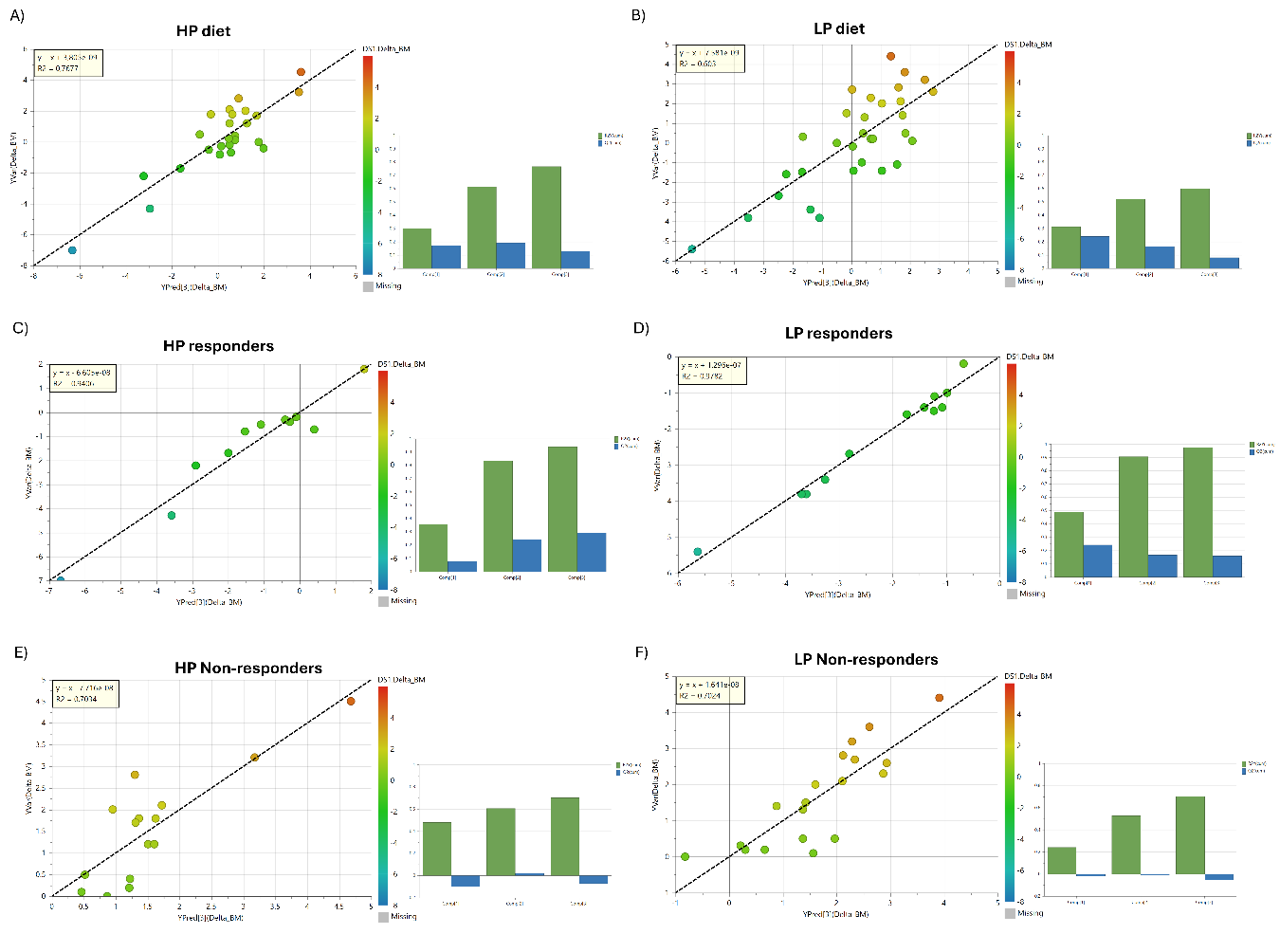


**Figure S6.** The observed versus predicted plot of PLS model with and their overview of the model validation; constructed to predict body mass (BM) changes from the urine metabolite profile measured at baseline considering HP diet (A), LP diet (B), responders to the HP diet (C), responders to the LP diet (D), non-responders to the HP diet (E), and non-responders to the LP diet (F).

**Table S1.** Urine metabolite concentrations in mM during interventions with HP and LP diet from samples collected at week 0 (wk-0) (baseline), at week 6 (mid-intervention) and at week 12 (wk-12) (endpoint).

|  |  | **Responders** | | **Non-responders** | |
| --- | --- | --- | --- | --- | --- |
|  |  | **HP** | **LP** | **HP** | **LP** |
| **1-Methylhistidine** | **Wk-0** | 0.0284 | 0.0344 | 0.0293 | 0.0232 |
|  | **Wk-6** | 0.0188 | 0.0160 | 0.0198 | 0.0242 |
|  | **Wk-12** | 0.0247 | 0.0341 | 0.0251 | 0.0235 |
| **2-Furoylglycine** | **Wk-0** | 0.0856 | 0.0667 | 0.0513 | 0.0527 |
|  | **Wk-6** | 0.0709 | 0.0320 | 0.0596 | 0.0480 |
|  | **Wk-12** | 0.0624 | 0.0456 | 0.0578 | 0.0470 |
| **3-Aminoisobutyrate** | **Wk-0** | 0.1887 | 0.3142 | 0.1190 | 0.0920 |
|  | **Wk-6** | 0.1026 | 0.0893 | 0.1784 | 0.1142 |
|  | **Wk-12** | 0.0751 | 0.1371 | 0.1110 | 0.0851 |
| **3-Hydroxybutyrate** | **Wk-0** | 0.0431 | 0.0590 | 0.0319 | 0.0280 |
|  | **Wk-6** | 0.0402 | 0.0353 | 0.0461 | 0.0329 |
|  | **Wk-12** | 0.0419 | 0.0282 | 0.0328 | 0.0232 |
| **Acetate** | **Wk-0** | 0.1184 | 0.0744 | 0.0743 | 0.0715 |
|  | **Wk-6** | 0.2179 | 0.0561 | 0.0851 | 0.0870 |
|  | **Wk-12** | 0.1147 | 0.0675 | 0.0731 | 0.0649 |
| **Acetoacetate** | **Wk-0** | 0.1508 | 0.2733 | 0.1051 | 0.0958 |
|  | **Wk-6** | 0.1523 | 0.1121 | 0.1259 | 0.0784 |
|  | **Wk-12** | 0.1611 | 0.1372 | 0.0936 | 0.0994 |
| **Acetone** | **Wk-0** | 0.0289 | 0.1199 | 0.0168 | 0.0161 |
|  | **Wk-6** | 0.0437 | 0.0228 | 0.0205 | 0.0135 |
|  | **Wk-12** | 0.0407 | 0.0233 | 0.0156 | 0.0176 |
| **Alanine** | **Wk-0** | 0.3049 | 0.2766 | 0.2005 | 0.2175 |
|  | **Wk-6** | 0.2451 | 0.2043 | 0.2153 | 0.2113 |
|  | **Wk-12** | 0.2300 | 0.1822 | 0.2935 | 0.2159 |
| **Allantoin** | **Wk-0** | 0.1568 | 0.1593 | 0.1103 | 0.1263 |
|  | **Wk-6** | 0.1306 | 0.1190 | 0.1278 | 0.1377 |
|  | **Wk-12** | 0.1401 | 0.1053 | 0.1258 | 0.1372 |
| **Betaine** | **Wk-0** | 0.1099 | 0.0818 | 0.0571 | 0.0619 |
|  | **Wk-6** | 0.0851 | 0.0959 | 0.0631 | 0.0593 |
|  | **Wk-12** | 0.0816 | 0.0670 | 0.0659 | 0.0872 |
| **Citrate** | **Wk-0** | 3.2600 | 2.7610 | 1.8710 | 2.2790 |
|  | **Wk-6** | 2.2250 | 2.3450 | 2.0160 | 2.1920 |
|  | **Wk-12** | 3.2090 | 2.4960 | 2.2690 | 2.2820 |
| **Creatine** | **Wk-0** | 0.3085 | 0.8297 | 0.4968 | 0.6623 |
|  | **Wk-6** | 0.3306 | 0.9736 | 0.8646 | 0.5299 |
|  | **Wk-12** | 0.2366 | 0.6755 | 0.4139 | 0.5693 |
| **Creatinine** | **Wk-0** | 9.9290 | 13.1700 | 6.5030 | 8.2280 |
|  | **Wk-6** | 7.9070 | 9.3000 | 7.9430 | 7.8560 |
|  | **Wk-12** | 8.8500 | 8.7540 | 7.4360 | 7.8470 |
| **Dimethylamine** | **Wk-0** | 0.3870 | 0.4741 | 0.2454 | 0.3103 |
|  | **Wk-6** | 0.3141 | 0.3071 | 0.2831 | 0.2825 |
|  | **Wk-12** | 0.3526 | 0.2946 | 0.2975 | 0.2821 |
| **Formate** | **Wk-0** | 0.2116 | 0.2560 | 0.1708 | 0.2052 |
|  | **Wk-6** | 0.1699 | 0.2089 | 0.2019 | 0.1841 |
|  | **Wk-12** | 0.1776 | 0.1875 | 0.1764 | 0.2082 |
| **Fumarate** | **Wk-0** | 0.0069 | 0.0061 | 0.0039 | 0.0042 |
|  | **Wk-6** | 0.0066 | 0.0039 | 0.0050 | 0.0042 |
|  | **Wk-12** | 0.0052 | 0.0036 | 0.0064 | 0.0036 |
| **Glucose** | **Wk-0** | 0.4131 | 0.5180 | 0.2897 | 0.3473 |
|  | **Wk-6** | 0.3014 | 0.4025 | 0.3490 | 0.2998 |
|  | **Wk-12** | 0.3647 | 0.3383 | 0.4132 | 0.3212 |
| **Glycine** | **Wk-0** | 2.2570 | 1.3360 | 1.0810 | 1.1550 |
|  | **Wk-6** | 1.5560 | 1.1530 | 1.3780 | 1.2850 |
|  | **Wk-12** | 1.7100 | 1.1740 | 1.4210 | 1.2890 |
| **Guanidoacetate** | **Wk-0** | 0.3708 | 0.3332 | 0.3410 | 0.2691 |
|  | **Wk-6** | 0.2962 | 0.3075 | 0.5833 | 0.2718 |
|  | **Wk-12** | 0.4051 | 0.5036 | 0.3153 | 0.2727 |
| **Hippurate** | **Wk-0** | 2.3480 | 1.7590 | 1.1770 | 1.3350 |
|  | **Wk-6** | 1.6720 | 0.8914 | 1.5050 | 1.4270 |
|  | **Wk-12** | 1.9750 | 1.2390 | 1.0840 | 1.1910 |
| **Indole-3-lactate** | **Wk-0** | 0.2164 | 0.2524 | 0.1493 | 0.1663 |
|  | **Wk-6** | 0.1609 | 0.2171 | 0.1650 | 0.1516 |
|  | **Wk-12** | 0.1867 | 0.2255 | 0.1451 | 0.1502 |
| **Lactate** | **Wk-0** | 0.2263 | 0.2241 | 0.1261 | 0.1537 |
|  | **Wk-6** | 0.1603 | 0.1282 | 0.1670 | 0.1523 |
|  | **Wk-12** | 0.2925 | 0.1502 | 0.3359 | 0.1317 |
| **N,N-Dimethylglycine** | **Wk-0** | 0.0280 | 0.0326 | 0.0261 | 0.0188 |
|  | **Wk-6** | 0.0155 | 0.0272 | 0.0176 | 0.0186 |
|  | **Wk-12** | 0.0291 | 0.0173 | 0.0163 | 0.0189 |
| **Phenylalanine** | **Wk-0** | 0.5737 | 0.3567 | 0.2997 | 0.3431 |
|  | **Wk-6** | 0.3472 | 0.3431 | 0.3483 | 0.3080 |
|  | **Wk-12** | 0.4198 | 0.3616 | 0.2861 | 0.2767 |
| **Succinate** | **Wk-0** | 0.0961 | 0.0410 | 0.0452 | 0.0489 |
|  | **Wk-6** | 0.0694 | 0.0407 | 0.0358 | 0.0434 |
|  | **Wk-12** | 0.0590 | 0.0453 | 0.0478 | 0.0441 |
| **Trigonelline** | **Wk-0** | 0.3381 | 0.1407 | 0.1431 | 0.1561 |
|  | **Wk-6** | 0.2115 | 0.0863 | 0.1622 | 0.1218 |
|  | **Wk-12** | 0.2804 | 0.1737 | 0.1558 | 0.1196 |
| **Trimethylamine** | **Wk-0** | 0.0439 | 0.0314 | 0.0258 | 0.0369 |
|  | **Wk-6** | 0.0302 | 0.0255 | 0.0275 | 0.0302 |
|  | **Wk-12** | 0.0213 | 0.0215 | 0.0253 | 0.0342 |
| **Trimethylamine N-oxide** | **Wk-0** | 0.5360 | 0.6057 | 0.2270 | 0.2711 |
|  | **Wk-6** | 0.3974 | 0.2597 | 0.2965 | 0.2452 |
|  | **Wk-12** | 0.2776 | 0.2963 | 0.3057 | 0.2406 |
| **Tyrosine** | **Wk-0** | 0.1252 | 0.0929 | 0.0774 | 0.0917 |
|  | **Wk-6** | 0.1042 | 0.0861 | 0.1027 | 0.0880 |
|  | **Wk-12** | 0.0994 | 0.0728 | 0.1090 | 0.1035 |
| **Urea** | **Wk-0** | 27.2200 | 26.8900 | 20.7700 | 22.7700 |
|  | **Wk-6** | 30.0500 | 24.8500 | 26.4500 | 20.6400 |
|  | **Wk-12** | 29.0100 | 21.8600 | 23.1700 | 22.1100 |
| **Valine** | **Wk-0** | 0.0372 | 0.0422 | 0.0260 | 0.0297 |
|  | **Wk-6** | 0.0353 | 0.0307 | 0.0323 | 0.0265 |
|  | **Wk-12** | 0.0369 | 0.0296 | 0.0339 | 0.0299 |
